# A novel integrated global coronal aligner helps prevent post-operative standing coronal imbalance in adult spinal deformity patients fused to pelvis: technical notes and preliminary results

**DOI:** 10.1186/s12891-021-04147-2

**Published:** 2021-03-26

**Authors:** Jiandang Zhang, Pengfei Chi, Junyao Cheng, Zheng Wang

**Affiliations:** Department of spine surgery, The Chinese PLA General Hospital, 28 Fuxing Rd, Beijing, 100853 China

**Keywords:** Adult spinal deformity, Integrated global coronal aligner, Coronal imbalance, Coronal balance difference

## Abstract

**Background:**

Chieving postoperative coronal balance in adult spinal deformity correction surgeries can be challenging. Even with T square rod technique, there were still some cases with good intraoperative coronal alignment but unsatisfactory post-operative standing coronal imbalance. Thus, the novel techniques to obtain global coronal balance are still in great needs. The purpose of this study was to describe a novel integrated global coronal aligner (IGCA) and evaluate its efficacy on avoidance of post-operative coronal imbalance in adult spinal deformity patients fused to pelvis.

**Methods:**

A detailed description of IGCA technique was presented. 52 ASD patients fused to pelvis were divided into two groups (IGCA group, *n* = 27; and non-IGCA group, *n* = 25) according to whether intraoperative IGCA was used or not. Preoperative demographics and postoperative outcomes were compared.

**Results:**

There were no significant differences regarding coronal balance difference (CBD) and imbalance/balance ratio between IGCA and non-IGCA groups preoperatively. After surgery, CBD in IGCA group was significantly improved from 24.7 ± 20.3 mm preoperatively to 12.6 ± 6.4 mm postoperatively (t = 3.185 *p* = 0.004), and imbalance/balance ratio decreased significantly from 55.6% (15/27) preoperatively to 11.1% (3/27) postoperatively (χ2 = 12.000, *p* = 0.001), while CBD and imbalance/balance ratio in non-IGCA group were not significantly improved. Compared to non-IGCA group, the amount of correction in CBD was significantly larger in IGCA group (t = 3.274, *P* = 0.002), and imbalance/balance ratio in IGCA group was significantly lowered (χ2 = 8.606 *p* = 0.003). Further logistic regression analysis revealed IGCA technique was associated with increased odds ratio for postoperative coronal balance (odds ratio: 7.385; 95% confidence interval 1.760–30.980; *P* = 0.006).

**Conclusions:**

The novel intraoperative IGCA technique could help improve CBD and reduce imbalance/balance ratio. It could help prevent post-operative coronal imbalance in adult spinal deformity patients fused to pelvis.

**Level of evidence:**

3

## Background

Adult spinal deformity (ASD) is a common healthcare concern that can frequently cause significant pain and disability, leading to decreased health related quality of daily life (HRQoL) [1, 2]. Given the proper indications, surgical treatments have been demonstrated to provide better clinical outcomes when compared to non-surgical treatments [3–5]. Achieving spinal balance is one of the major purposes of corrective spinal deformity surgeries. In the past decade, more emphasis has been laid on the restoration of sagittal balance due to its great impact on HRQoL, however, more and more studies have recently shown that postoperative coronal imbalance can also negatively affect quality of life in adults [6–8].

Unfortunately, achieving postoperative coronal balance in long deformity corrections can be challenging [9]. T square rod has been used to help correct coronal malalignment and showed positive effect on the amount of spine coronal malalignment correction [10]. However, T square rod only assesses spinal coronal alignment, which is only part of global coronal alignment. Even with T square rod technique, we still came across some cases with good intraoperative coronal alignment but unsatisfactory postoperative coronal imbalance while in standing position or ambulatory status. For deformity patients that need to be extensively fused to the pelvis, the spine has little ability to compensate once coronal imbalance occurs. Thus, novel techniques to obtain global coronal balance are still in great needs.

In a normal healthy standing person, ideally, C7 plumb line would overlap central sacral vertical line (CSVL), and pass through gluteal cleft, midpoints between bilateral knees/ankles/heels. According to this common knowledge, we developed an “integrated global coronal aligner (IGCA)” technique by also utilizing physical landmarks of lower body part in addition to use of inverted cross device (a modified T square rod) during surgery. The purpose of this study was (1) to describe the IGCA technique, and (2) to assess its efficacy on avoidance of postoperative coronal imbalance in posterior spinal deformity correction surgeries with five or more levels fused to the pelvis.

## Methods

### Patient population

Approval by Ethical Committee of our hospital was obtained prior to this study. We collected data of ASD patients who underwent primary spinal correction and fusion surgeries through posterior-only approach with age at surgery > 45 years in one institution between January 2016 and May 2019. All procedures were performed by the same surgical team. Exclusion criteria included fusion levels < 5, congenital deformity, post-traumatic deformity, neuromuscular disease, spinal tumor, Pott’s deformity, pelvic deformity, absolute discrepancy of leg length > 20 mm and lower instrumented vertebra at L5 or above. Eventually, 52 ASD patients fused to pelvis were enrolled in this study (8 males, 44 females; average age at surgery: 64.3 ± 7.2 yr). Based on whether intraoperative IGCA was used or not, these 52 patients were further divided into two groups (IGCA group, *n* = 27; and non-IGCA group, *n* = 25).

### Surgical techniques

**Fig. 1:**
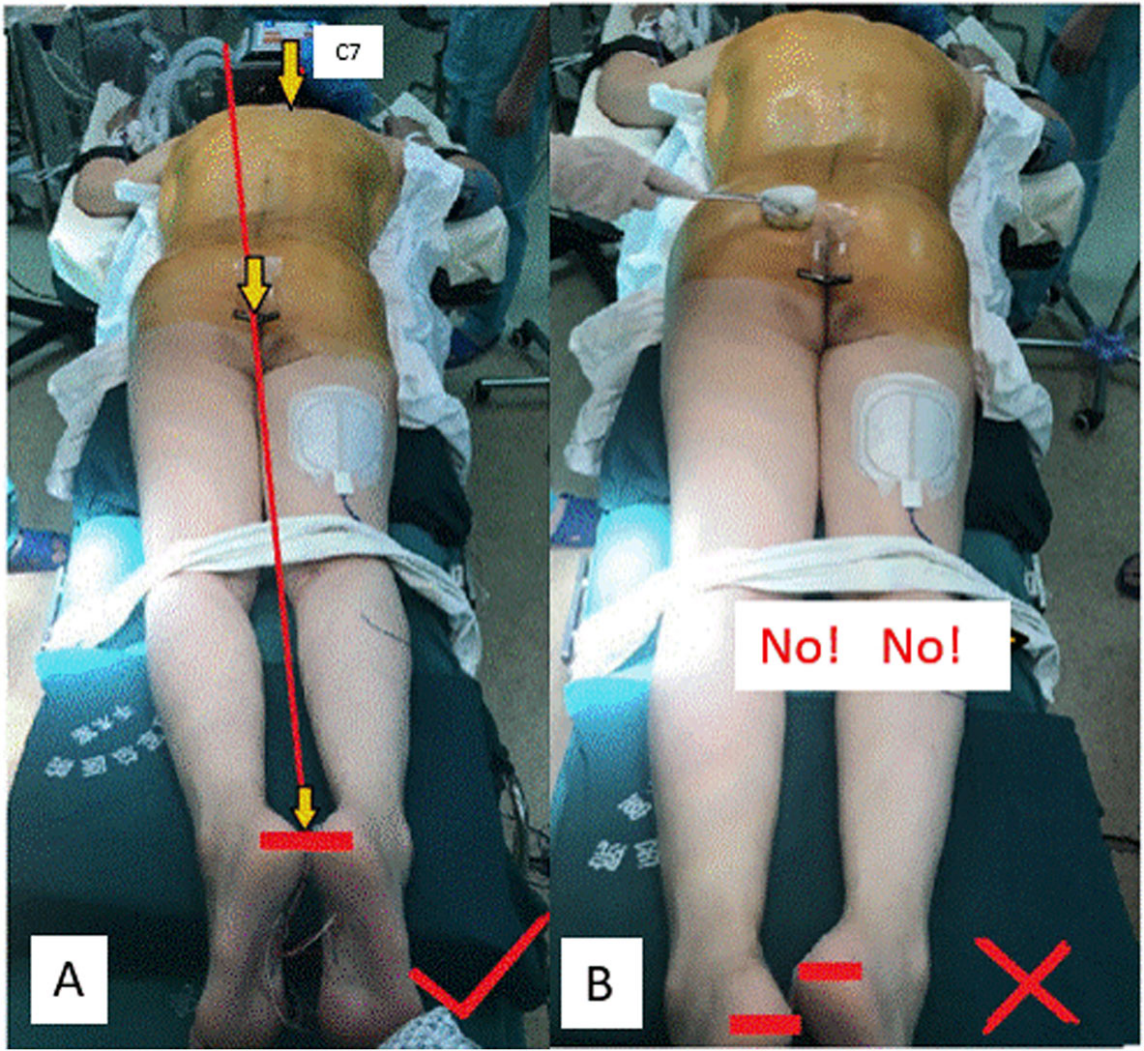
**a** Establishing lower body part aligner. **b** A common mistake during patient’s positioning

**Fig. 2:**
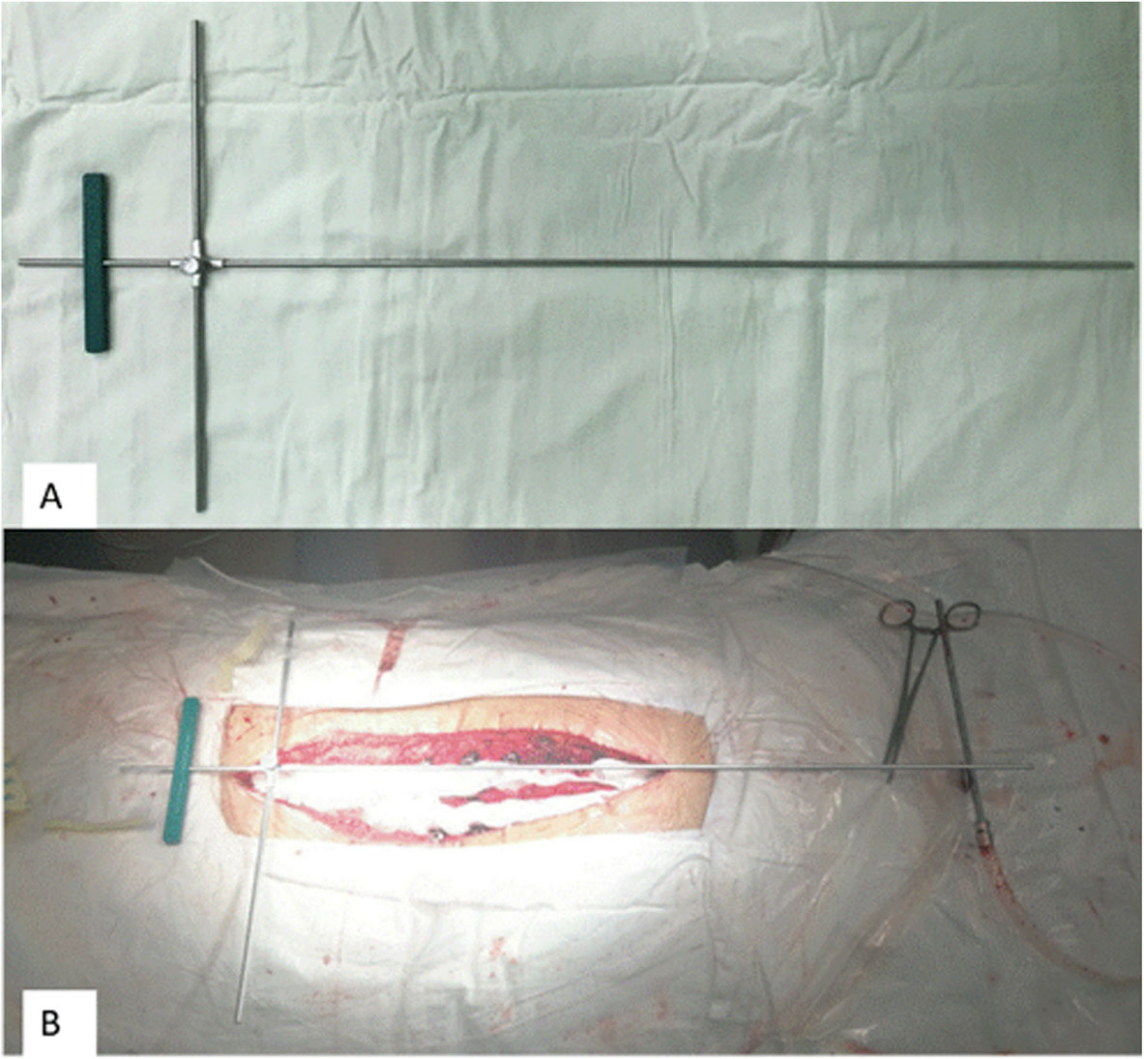
**a** Inverted cross device (upper body part aligner) and (**b**) being aligned on a patient

IGCA consists of 2 parts: (1), lower body part aligner: it is an imaginary line made up of physical surface landmarks of lower body part such as the midpoints between two symmetrical heels/ankles/knees, passing through gluteal cleft, this line overlaps CSVL (Fig. 1a); and (2), upper body part aligner, which is served by an inverted cross device. This device is composed of one shorter vertical limb, one longer vertical limb and two horizontal limbs of equal length (Fig. 2a), the green plastic bar is a scaled marker, which makes the shorter vertical limb overlay CSVL more easily intraoperatively. The longer vertical limb is telescoped, which can slide inward or outward depending on patient’s height. When the shorter vertical limb overlays CSVL, ideally, the longer vertical limb would pass across C7 center.

The technical notes of IGCA were described as below:

#### Anesthesia, positioning and establishing lower body part aligner

After general endotracheal anesthesia performed, the patient was placed in a prone position. First, the heel bottoms were adjusted to be flush with each other, then bilateral ankles and knees were made symmetrical and at the same level, respectively. Thus, the midpoints between two heels/two ankles/two knees, and gluteal cleft were made on the same line, which is the lower body part aligner (Fig. 1a). To establish this lower body part aligner, upper body part (body trunk) of severe spinal deformity patients was usually placed partially off the operation table and supported by an added frame. In contrast to priority of upper body part positioning in common spinal surgeries, positioning of lower body part was prioritized in this technique of ours, which set a basis for spinal deformity to be corrected. The common mistake is to only focus on positioning of the upper body part and ignore the presence of iatrogenic discrepancy of leg length (Fig. 1b), which might lead to postoperative coronal imbalance in standing position.

#### Upper body part aligner- intraoperative inverted cross device

**Fig. 3:**
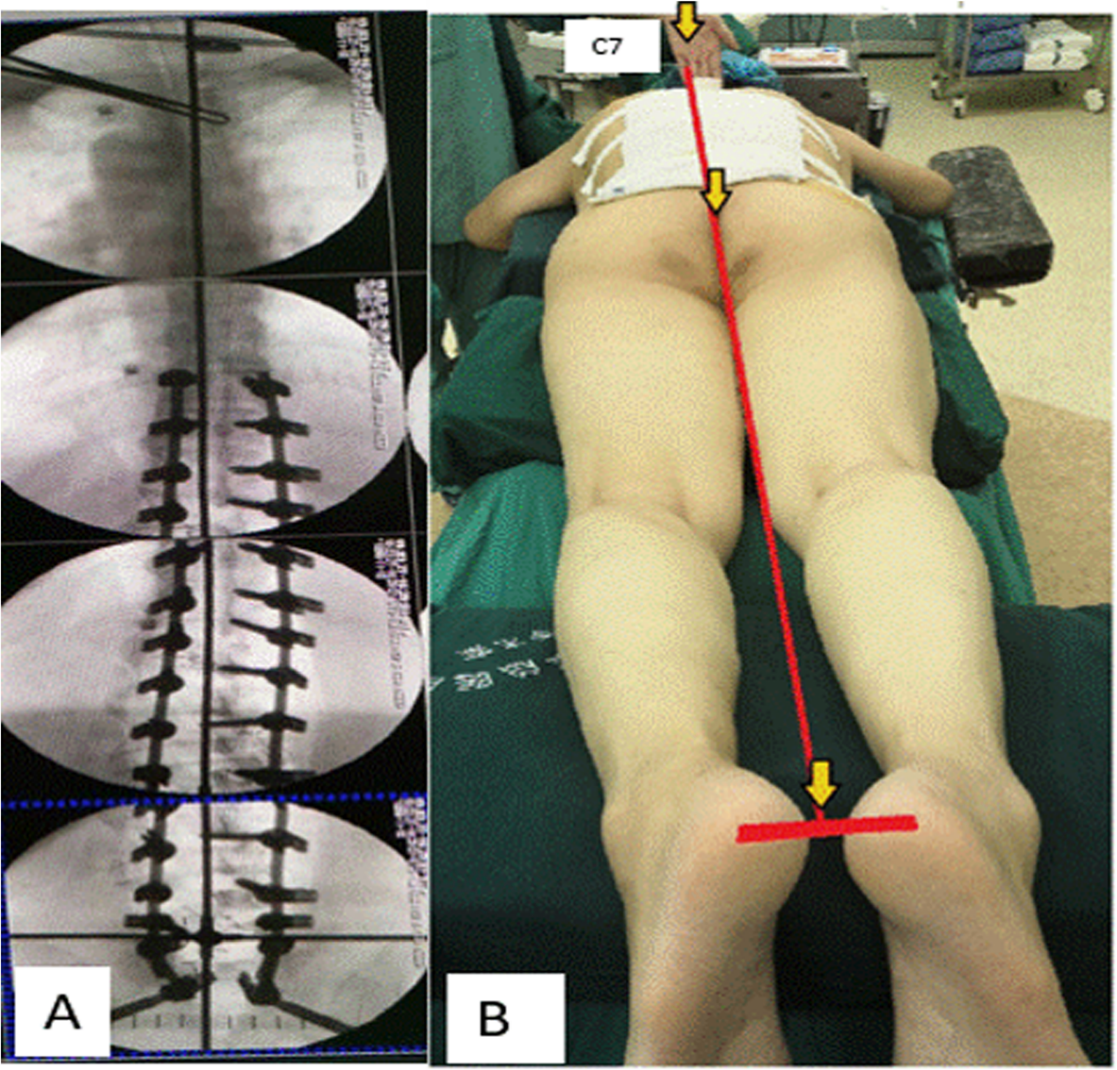
**a** Checking fluoroscopically if longer vertical limb passed across C7. **b** A perfect line passing through physical landmarks after IGCA technique. IGCA: integrated global coronal aligner

The inverted cross device was used at the final steps of the instrumentation. After the deformity was initially corrected, the inverted cross device was to be aligned on a patient (Fig. 2b). Its horizontal limbs were placed on the pelvis to align with supra-iliac line and shorter vertical limb in line with CSVL by using C arm. The longer vertical limb was then imaged fluoroscopically to check if it passed through C7 body (Fig. 3a). If it did, a well-balanced body would be obtained (Fig. 3b); If it did not, further maneuvers such as in situ coronal bending of the rods, compression, and distraction were performed to improve spinal deformity correction until C7 body was crossed.

### Radiographic evaluation

**Fig. 4:**
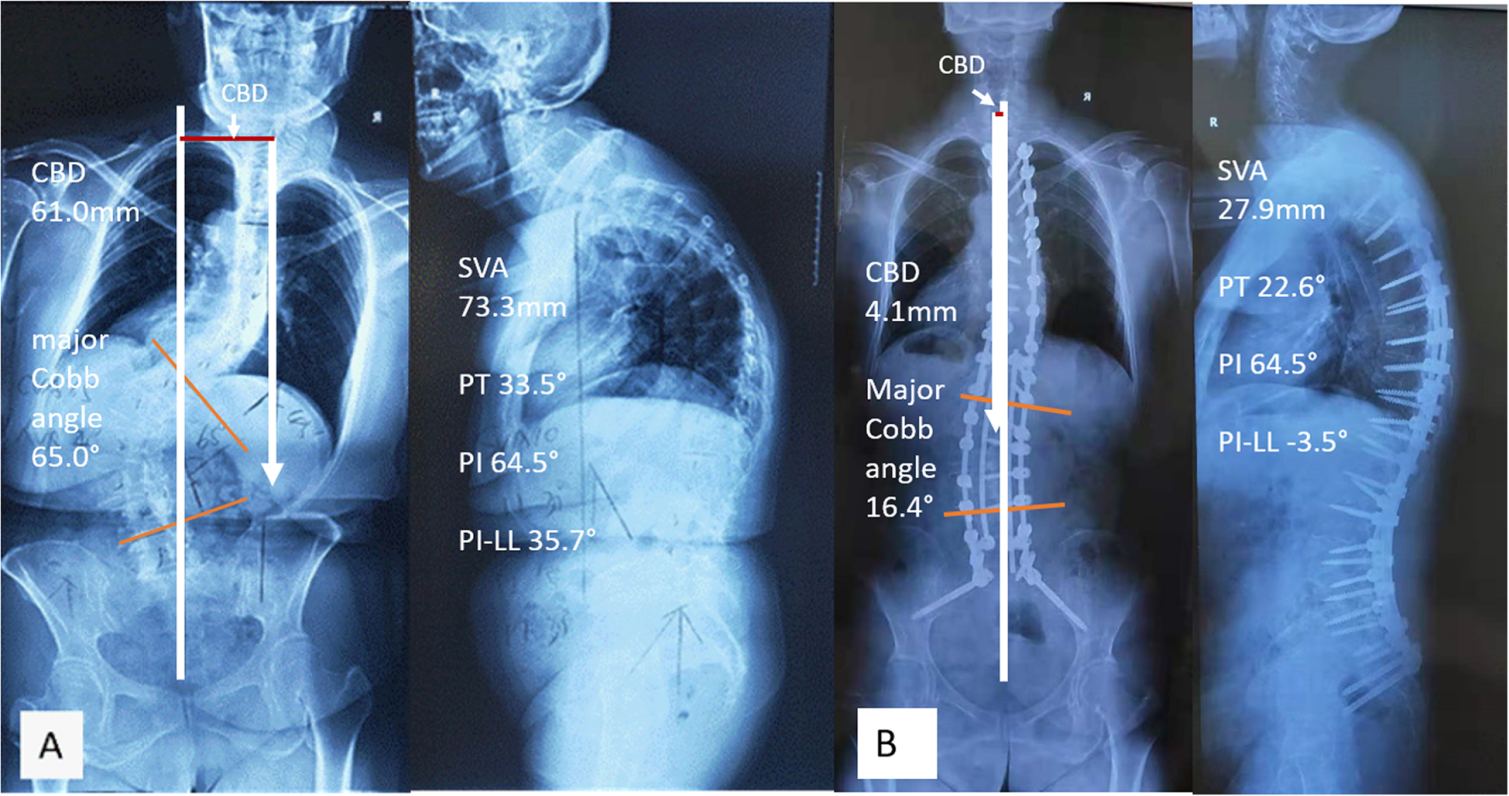
classic case, a 52-year-old lady. Pre-operative (**a**) and post-operative (**b**) standing posteroanterior and lateral radiographs after IGCA technique. IGCA: integrated global coronal aligner

Full-spine standing posteroanterior and lateral radiographs were analyzed preoperatively and post-op 2 weeks or at hospital discharge (Fig. 4a, b). The measurements were done using Surgimap (version 2.2.15; Spine Software, New York, NY) by two independent researchers and the mean values were collected for analysis. The following parameters were measured on the coronal plane: (1) coronal balance distance (CBD), defined as the horizontal distance between the C7 plumb line (C7 PL) and CSVL, C7 PL shifted to the right was defined as positive, and to the left as negative; (2) major Cobb angle. Right curve was defined as positive, left curve as negative; (3) the correction in CBD was defined as ΔCBD, ΔCBD = preoperative CBD- postoperative CBD; (4) the correction in major Cobb angle was defined as Δ major Cobb angle, Δ major Cobb angle = preoperative major Cobb angle- postoperative major Cobb angle. Sagittal parameters included: (1) thoracic kyphosis (TK), the angle between the inferior endplate of T5 and T12, kyphosis was defined as positive, and lordosis as negative; (2) pelvic tilt (PT), a pelvic positional parameter; (3) pelvic incidence minus lumbar lordosis, (PI-LL); (4) sagittal vertical axis (SVA), the distance between C7 PL and posterosuperior corner of S1. (5) corrections in sagittal parameters such as Δ thoracic kyphosis, Δ PT, Δ PI-LL, and Δ SVA, they were defined in the same way as corrections in coronal parameters.

Imbalance/balance ratio, instrumented levels and distribution of upper instrumented vertebra (UIV) were compared as well. Because spinal osteotomies were performed in all patients (mean levels 3.3 ± 0.9, range 2–5 levels), including Schwab grade I in 5 patients, grade II in 47 patients, osteotomy levels and osteotomy grades between two groups were also analyzed. Coronal imbalance was defined as CBD greater than 20 mm.

### Statistical analysis

Prior to the statistical analysis, the values of CBD and major Cobb angle were converted to absolute values. Descriptive statistics were performed to determine means and standard deviations. Continuous variables were compared between groups using independent samples t test, within groups using paired samples t test. Categorical variables were compared using Chi-square analysis. Binary logistic regression analysis was further performed to estimate odds ratio for postoperative coronal imbalance. In binary logistic regression analysis, the non-IGCA was coded as “0”, and IGCA as “1”. The statistical analysis was performed using SPSS computer software (version 24; SPSS, Chicago, IL, USA). A *p*-value of < 0.05 was considered as statistically significant.

## Results

### Patients’ characteristics of IGCA and non-IGCA patients

Table 1 shows the patients’ demographics for the IGCA and non-IGCA groups. There were no significant differences regarding sex, age at surgery, instrumented levels, distribution of UIV, osteotomy grades and osteotomy levels between IGCA and non-IGCA groups (Table 1).

**Table 1:** Patient characteristics between IGCA and non-IGCA groups

	IGCA	Non-IGCA	t/χ2 value	*P* value
Patient No.	27	25		
Sex (M:F)	2:25	6:19	2.745	0.098#
Age at surgery (yr)	63.5 ± 6.4	65.2 ± 8.0	−0.863	0.392*
Instrumented levels	9.6 ± 2.2	8.8 ± 2.8	1.142	0.259*
UIV (T10 or above: below)	21:6	15:10	1.926	0.165#
Osteotomy grades	1.9 ± 0.3	1.9 ± 0.3	0.552	0.583*
Osteotomy levels	3.5 ± 1.0	3.1 ± 0.8	1.327	0.191*

Results are given as the number or the mean ± standard deviation (SD) unless otherwise stated

*Independent t test

# Chi-square test

*IGCA* indicates integrated global coronal aligner, *UIV* Upper instrumented vertebra

### Comparison of radiographic parameters between IGCA and non-IGCA groups

Preoperatively, there were no significant differences regarding CBD, major Cobb angle and imbalance/balance ratio between IGCA and non-IGCA groups (Table 2). After surgery, the CBD in IGCA group was significantly improved from 24.7 ± 20.3 mm preoperatively to 12.6 ± 6.4 mm postoperatively (t = 3.185, *p* = 0.004), and the imbalance/balance ratio was reduced significantly from 55.6% (15/27) preoperatively to 11.1% (3/27) postoperatively (χ2 = 12.000, *p* = 0.001). On the contrary, the CBD in non-IGCA group was not improved (17.4 ± 15.1 vs. 18.3 ± 11.4, t = 0.327 *p* = 0.747)), and the imbalance/balance ratio frustratingly increased from 32% (8/25) preoperatively to 48% (12/25) postoperatively (χ2 = 1.333, *p* = 0.248). Compared to the non-IGCA group, the amount of correction in CBD (CBD) was significantly larger in the IGCA group (t = 3.274, *P* = 0.002), and the imbalance/balance ratio in IGCA group was significantly lowered (χ2 = 8.606 *p* = 0.003) (Table 2). The major Cobb angle in addition to sagittal parameters (SVA, TK, PT, PI-LL) and their changes exhibited no significant differences between IGCA and non-IGCA groups (Tables 2, 3).

**Table 2:** Pre- and post-operative coronal parameters and their changes between two groups

Parameters	IGCA	Non-IGCA	t/χ2 value	*P* value
No. of patients	27	25		
**CBD**				
Preoperative	24.7 ± 20.3	17.4 ± 15.1	1.466	0.149^*^
Postoperative	12.6 ± 6.4	18.3 ± 11.4	−1.364	0.179^*^
**Major Cobb angle**				
Preoperative	25.4 ± 14.1	23.2 ± 13.4	0.572	0.570^*^
Postoperative	8.8 ± 7.5	9.1 ± 6.9	−0.196	0.845^*^
Δ CBD	13.9 ± 18.6	−1.3 ± 14.2	3.274	0.002^*^
Δ major Cobb angle	16.6 ± 10.8	14.1 ± 9.7	0.904	0.370
**Imbalance: balance**				
Preoperative	15:12	8:17	2.920	0.087^**#**^
Postoperative	3:24	12:13	8.606	**0.003**^**#**^

Results are given as the number or the mean ± standard deviation (SD) unless otherwise stated

*IGCA* integrated global coronal aligner, *CBD* Coronal balance difference, *PI-LL* Pelvic incidence minus lumbar lordosis

^*^independent samples of t test

^#^Chi-square test

**Table 3:** Comparison of pre- and postoperative sagittal parameters and their changes between two groups (independent t test)

	IGCA	Non-IGCA	t/χ2 value	*P* value
Patient No.	27	25		
**Thoracic kyphosis**				
Preoperative	12.7 ± 13.3	14.1 ± 9.1	−0.097	0.923
Postoperative	20.8 ± 9.3	21.5 ± 10.1	−0.263	0.793
**PI-LL**				
Preoperative	26.1 ± 17.9	24.3 ± 19.9	0.333	0.740
Postoperative	6.5 ± 10.1	8.3 ± 10.2	−1.708	0.694
**Pelvic tilt**				
Preoperative	27.6 ± 13.4	26.1 ± 11.5	0.424	0.673
Postoperative	16.9 ± 9.6	18.6 ± 9.4	−0.650	0.518
**SVA**				
Preoperative	73.5 ± 41.9	82.1 ± 56.0	−0.636	0.528
Postoperative	32.1 ± 20.4	30.7 ± 16.6	0.270	0.788
Δ thoracic kyphosis	‘-8.1 ± 8.9	‘-6.5 ± 10.6	−0.577	0.567
Δ PI-LL	19.8 ± 13.7	14.3 ± 13.6	1.441	0.156
Δ pelvic tilt	10.8 ± 10.1	7.6 ± 8.8	1.199	0.236
Δ SVA	41.4 ± 33.9	51.4 ± 48.4	−0.875	0.386

Results are given as the number or the mean ± standard deviation (SD) unless otherwise stated

*IGCA* Integrated global coronal aligner, *PI-LL* Pelvic incidence minus lumbar lordosis, *SVA* Sagittal vertical axis

### Regression analysis

Since Chi-square test results showed that IGCA technique could significantly reduce imbalance/balance ratio when compared to non-IGCA group, further binary logistic regression analysis was performed to estimate odds ratios for postoperative coronal balance when IGCA was used, and the results of regression analysis revealed IGCA technique was associated with increased odds ratio for postoperative coronal balance (odds ratio: 7.385; 95% confidence interval 1.760–30.980; *P* = 0.006).

## Discussion

The current study demonstrated significant improvement in CBD and decrease in imbalance/balance ratio after IGCA technique was used intraoperatively.

In the non- IGCA group, the CBD was not improved after surgery, and the imbalance/balance ratio frustratingly increased from 32% (8/25) preoperatively to 48% (12/25) postoperatively. It is not rare that corrective surgery for ASD fails to correct coronal malalignment or even worsens it in the literature. Ploumis et al. retrospectively analyzed 54 ASD patients after long fusions with minimum 2 years follow-up and found the of coronal malalignment did not improve from the preoperative, regardless of improved sagittal malalignment by surgery [9]. Kurra S et al. reported that there was no significant improvement in coronal malalignment in non-T square rod patients postoperatively versus preoperatively [10].

On the contrary, in the IGCA group, the imbalance/balance ratio decreased significantly from 55.6% (15/27) preoperatively to 11.1% (3/27) postoperatively, and the CBD was significantly improved. Similar to our results, Kurra S et al. reported significant coronal malalignment improvement in T square rod used patients from the preoperative to the postoperative [10]. These studies highlighted the importance of intraoperative coronal aligner.

An interesting finding in our results is that significant difference regarding correction of major Cobb angels (Δ major Cobb angle) were not seen in IGCA group when compared to non-IGCA group (t = 0.904, *P* = 0.370), although post-operative CBD was significantly improved by using IGCA. The possible explanation might be due to multi-level spinal osteotomies performed in all patients (mean levels 3.3), which helped achieve satisfactory curve correction in both groups. On the other hand, CBD reflects global coronal balance, whereas major Cobb angle is a regional parameter. In addition to major Cobb angle, there are other factors influencing the global balance. It suggested that use of global coronal aligner to achieve global balance be more important than use of upper body part aligner only.

T square rod is an effective tool to help correct coronal malalignment in spinal deformity patients reported by Kurra [10] and Andras [11]. But T square rod technique only considers upper body part alignment and does not take the whole body into consideration. In our practice, we still came across postoperative standing coronal imbalance even when intraoperative T square rod was used. Also, in Kurra’s own report [10], they still had patients with postoperative CBD as big as 40 mm in T square rod used patients, although prevalence of coronal imbalance in T square rod used patients postoperatively was not reported in their paper.

To overcome this defect, we created an intraoperative IGCA. With IGCA technique, Imbalance/balance ratio decreased significantly, and CBD were significantly improved when compared to non-IGCA group; further binary logistic regression revealed IGCA technique was significantly associated with increased odds ratio for postoperative coronal balance. The IGCA technique consisted of lower body part aligner and upper body part aligner (inverted cross device). When establishing lower body part aligner before exposure, one common mistake during patient’s positioning was to only focus on positioning of the upper body part and ignore the presence of iatrogenic discrepancy of leg length. This might result in postoperative standing coronal imbalance. The inverted cross device is a modified T square rod, a green plastic scaled marker attached makes it easier to align the shorter vertical limb with CSVL. Several ways have been reported to make horizontal limbs parallel the pelvis such as use of supra-iliac line (the line connecting iliac crests), supra-acetabular line or femoral head center line (the line connecting two femoral head centers) overlapped by horizontal limbs [10, 11]. Recently, Hey HWD et al. [12] reported supra-acetabular line was better than supra-iliac line in determining coronal balance. But supra-acetabular line is still not ideal due to its unsatisfactory predictive value, better intraoperative markers for achieving coronal balance still needs to be identified.

The limitations in our study must be mentioned: 1, Functional scores such as SRS-22 or Oswestry disability index (ODI) scores were not involved in this study; 2, this was a relatively small sample-sized study; 3, this study was carried out in one single institution and involved one surgical team. A multi-center and multiple surgeons involved study with high number of patients may be more powerful to assess the efficacy of this technique. 4, The current study only reported the efficacy of IGCA technique on the avoidance of immediate postoperative coronal imbalance in spinal deformity correction patients fused to the pelvis. Although those patients had little capacity to compensate once postoperative coronal imbalance happens, further long-term followup study is needed. Despite the above-mentioned limitations, our study still demonstrated that IGCA technique could significantly improve CBD correction and reduce imbalance/balance ratio in ASD patients fused to pelvis.

## Conclusion

The IGCA technique could significantly improve CBD correction and reduce imbalance/balance ratio. It might help prevent post-operative coronal imbalance in ASD patients fused to pelvis.

## Data Availability

The datasets generated and/or analyzed during the current study are not. publicly available but are available from the corresponding author on. reasonable request.

## Abbreviations

IGCA: Integrated global coronal aligner
CBD: Coronal balance difference
ASD: Adult spinal deformity
C7 PL: C7 plumb line
UIV: Upper instrumented vertebra
CSVL: Central sacral vertical line
SVA: Sagittal vertical axis
TK: Thoracic kyphosis
PT: Pelvic tilt
PI-LL: Pelvic incidence- lumbar lordosis
ODI: Oswestry disability index
